# Androgen receptor regulates SRC expression through microRNA-203

**DOI:** 10.18632/oncotarget.8366

**Published:** 2016-03-25

**Authors:** Man Kit Siu, Wei-Yu Chen, Hong-Yuan Tsai, Hsiu-Lien Yeh, Juan Juan Yin, Shih-Yang Liu, Yen-Nien Liu

**Affiliations:** ^1^ Ph.D. Program for Cancer Biology and Drug Discovery, College of Medical Science and Technology, Taipei Medical University and Academia Sinica, Taipei, Taiwan; ^2^ Graduate Institute of Cancer Biology and Drug Discovery, College of Medical Science and Technology, Taipei Medical University, Taipei, Taiwan; ^3^ Department of Anesthesiology, Wan Fang Hospital, Taipei Medical University, Taipei, Taiwan; ^4^ Department of Pathology, Wan Fang Hospital, Taipei Medical University, Taipei, Taiwan; ^5^ Department of Pathology, School of Medicine, College of Medicine, Taipei Medical University, Taipei, Taiwan; ^6^ Institute of Information System and Applications, National Tsing Hua University, Hsinchu, Taiwan; ^7^ Cell and Cancer Biology Branch, National Cancer Institute, National Institutes of Health, Bethesda, MD, USA; ^8^ Department of Andrology, Pingtan Hospital of Traditional Chinese Medicine, Fujian, China

**Keywords:** androgen receptor (AR), microRNA (miR)-203, prostate cancer (PCa), SRC

## Abstract

The SRC kinase has pivotal roles in multiple developmental processes and in tumor progression. An inverse relationship has been observed between androgen receptor (AR) activity and SRC signaling in advanced prostate cancer (PCa); however, the modulation of AR/SRC crosstalk that leads to metastatic PCa is unclear. Here, we showed that patients with high SRC levels displayed correspondingly low canonical AR gene signatures. Our results demonstrated that activated AR induced miR-203 and reduced SRC levels in PCa model systems. miR-203 directly binds to the 3′ UTR of *SRC* and regulates the stability of SRC mRNA upon AR activation. Moreover, we found that progressive PCa cell migration and growth were associated with a decrease in AR-regulated miR-203 and an increase in SRC. Relationships among AR, miR-203, and SRC were also confirmed in clinical datasets and specimens. We suggest that the induction of SRC results in increased PCa metastasis that is linked to the dysregulation of the AR signaling pathway through the inactivation of miR-203.

## INTRODUCTION

Prostate cancer (PCa) is the most common male malignancy and the second leading cause of cancer deaths among men in the developed world [1]. Although significant gains have been made in managing early phases of PCa, most tumors progress to a hormone-independent metastatic disease, with limited therapeutic options and poor prognoses [2]. The survival of malignant tumors that arise from the prostate gland is dependent on the androgen receptor (AR), a classical nuclear steroid receptor that binds androgen and activates gene transcription [3, 4]. This dependence on androgen is often therapeutically exploited; patients presenting with metastatic disease are treated with anti-androgen therapies that effectively lower circulating androgen levels and cause tumor regression. However, patients typically relapse within 1–2 years and develop castration-resistant disease, in which tumors no longer respond to androgen-ablation therapy [5]. Understanding the AR signaling-dependent molecular controls that induce PCa progression and metastasis is key to developing better therapeutic and diagnostic tools for this disease.

SRC kinase regulates several upstream molecular signaling components, including numerous G-protein-coupled receptors, integrins, and receptor tyrosine kinases. SRC kinase also regulates numerous cell-signaling pathways that are important for cancer cell survival, proliferation, invasion, migration, and angiogenesis [6, 7]. Clinical observations have shown that the expression of AR and SRC is often elevated in castration-resistant PCa (CRPC) [8], and several studies have provided further evidence that SRC kinase can interact with AR signaling pathways [9–11]. AR has also been reported to undergo tyrosine phosphorylation and activation by SRC kinase [12]. Importantly, histopathological analyses of clinical samples demonstrated that increasing SRC activity was correlated with decreasing AR activity [13]. Our recent work supported an inverse AR and SRC regulatory network in which the loss of an AR-dependent transcriptional regulatory link with activated SRC promoted PCa bone metastasis [14]. We hypothesized that AR may act on SRC by affecting the expression of microRNAs (miRs) to post-transcriptionally regulate SRC expression in PCa cells.

Post-transcriptional regulation is increasingly believed to play an essential role in cancer development and progression [15]. miRs are a class of endogenous small RNA molecules of approximately 22 nucleotides [16] that govern diverse cellular activities, including proliferation, apoptosis, differentiation, development, and tumorigenesis, by targeting the RNA-induced silencing complex in the 3′-untranslated region (UTR) of target messenger (m)RNAs [17, 18]. miR-203 was identified as a stemness-inhibiting miR that is highly expressed in the epidermis, where it targets the α and β isoforms of TP63 to promote epidermal differentiation [19, 20]. In addition to its role in normal epithelial biology, miR-203 was also shown to be aberrantly expressed in several types of human cancers, including PCa [21–27]. Importantly, miR-203 was proposed as an “anti-metastatic” miR in PCa that acts at multiple steps of the PCa metastatic cascade by repressing a cohort of prometastatic targets [25, 27]. Moreover, miR-203 overexpression inhibits PCa cell invasion by targeting the 3′UTR of the polycomb repressive complex (PRC) 1 gene mRNA [28]. Our previous work demonstrated that activated epidermal growth factor receptor (EGFR) signaling induced Snail/PRC expression and downregulated *has-mir-203* stem-loop transactivation, thereby silencing miR-203 expression [29]. However, the androgen-dependent regulation of miR-203 remains to be investigated.

We examined the expression of AR and SRC in PCa samples, as well as their associations with miR-203. Relationships among AR, miR-203, and SRC were validated using two PCa databases. The regulatory mechanism was further confirmed by a promoter reporter assay and a 3′UTR luciferase assay. Treatment with a miR-203 inhibitor induced PCa malignancy, whereas restoration of miR-203 compromised this transformation. Collectively, our study revealed that the post-transcriptional regulation of SRC by AR-regulated miR-203 contributes to deregulated cell growth and motility in PCa.

## RESULTS

### Induced miR-203 expression is associated with activated AR signaling

**Figure 1:**
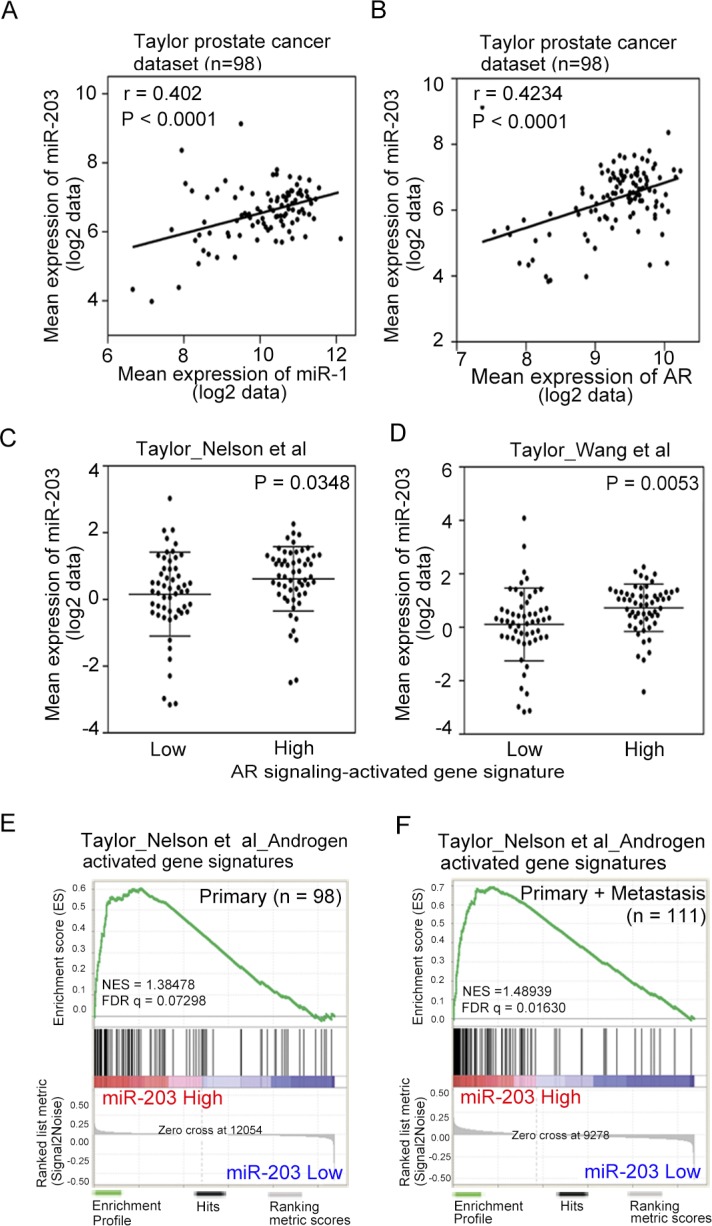
Induced miR-203 expression is associated with activated androgen receptor (AR) signaling (**A** and **B**) Pearson correlation coefficient analysis of mean miR-203 versus mean miR-1 (A) and AR mRNA (B) expression in primary PCa samples of the Taylor PCa dataset (*n* = 98). Significance was determined by the Gaussian population (Pearson) test. (**C** and **D**) Mean expression of miR-203 in the Taylor PCa dataset (*n* = 98) relative to gene sets of Nelson et al. (C) and Wang et al. (D), the expression of which increased or decreased with AR signaling in PCa tissues. Statistical significance was determined by Student's *t*-test. (**E** and **F**) Gene set enrichment analysis (GSEA) of the Taylor PCa dataset of primary (*n* = 98) (E) and combined primary and metastatic (*n* = 111) (F) tumors showing enrichment of miR-203 expression in the gene set of Nelson et al., the expression of which was associated with patients with increased AR signaling. NES, normalized enrichment score; FDR, false discovery rate.

Our previous study suggested that the loss of androgen-activated miR-1 is one of the mechanistic links with high SRC output, which promotes prostate metastatic phenotypes [14]. We also proposed that miR-203 is a tumor-suppressive miR in PCa and that miR-203 suppresses experimental bone metastasis [29]. To examine the relationship between miR-203 and miR-1 in PCa progression, we conducted a correlation analysis and found that the mean expression of miR-203 was significantly positively correlated with miR-1 expression in human prostate tissues using the Taylor PCa dataset [30] from the Memorial Sloan Kettering Cancer Center (MSKCC), which includes gene expression data from 98 primary tumor tissue specimens (Figure 1A). To investigate the connection between AR expression and miR-203 levels, the positive correlation between AR and miR-203 levels was confirmed by statistical analyses in clinical prostate samples from the Taylor PCa dataset (Figure 1B).

We hypothesized that AR signaling plays an important role in activating miR-203 in PCa. To address this question, we analyzed the relationships between miR-203 expression and two gene signatures [31, 32] that reflect activated AR signaling pathway components in the Taylor PCa dataset using a z-score analysis. High levels of miR-203 expression were positively associated with high expression levels of AR signaling-activated genes in those samples (Figure 1C, 1D). Similar results were obtained using a different database downloaded from the Cancer Genome Atlas (TCGA), which includes gene expression data from 372 primary PCa samples (TCGA web site; Supplementary Figure S1A, S1B). We further investigated the correlation between AR signaling and miR-203 levels and observed that increased levels of miR-203 expression were strongly associated with the induced expression of two different AR pathway gene signatures [31, 32] according to a gene set enrichment analysis (GSEA) of the Taylor PCa dataset (Figure 1E, 1F, and Supplementary Figure S1C, S1D).

To investigate whether miR-203 expression levels are related to AR output, we analyzed miR-203 expression levels and correlative mRNAs in the Taylor PCa dataset. We divided the specimens into two groups with ‘low’ and ‘high’ AR expression based on the mean mRNA expression and confirmed that tumors expressing higher levels of AR displayed significantly greater miR-203 levels (Supplementary Figure S1E). Similarly, we divided specimens into two groups with ‘low’ and ‘high’ miR-203 expression, and higher levels of miR-203 were found in tissues with high AR expression (Supplementary Figure S1F). These results are consistent with our proposed mechanism in which miR-203 function is stimulated by AR expression levels partly through regulation by activated AR signaling.

### miR-203 levels are directly activated by AR binding to the primary miR-203 promoter

**Figure 2:**
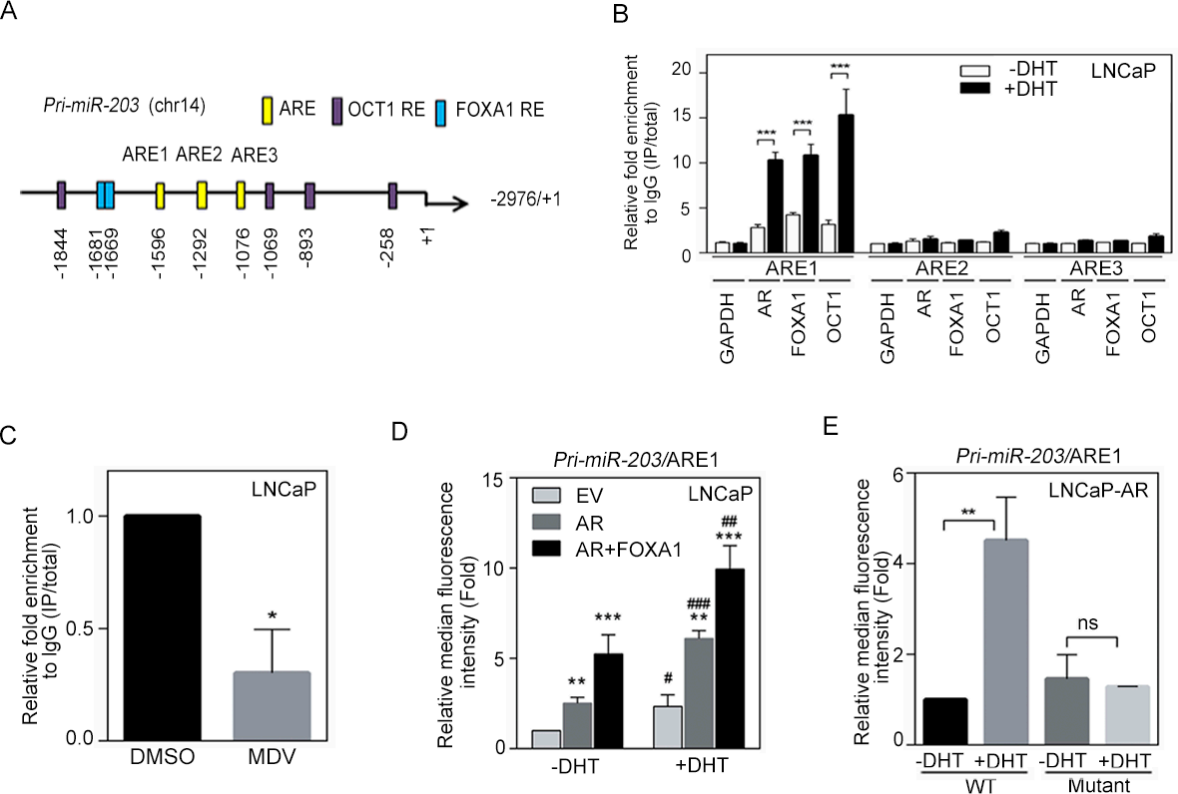
miR-203 levels are directly and positively regulated by androgen receptor (AR) binding to the *pri-miR-203* promoter (**A**) Schematic of the predicted AR, FOXA1, and OCT1 response elements (REs) in promoter reporter constructs of human primary miR-203 (*pri-miR-203*). (**B**) Chromatin immunoprecipitation (ChIP) assay showing the binding of AR, FOXA1, and OCT1 to the *pri-miR-203* promoter in LNCaP cells after 24 h dihydrotestosterone (DHT) treatment. Enrichment of each protein at each site is given as a percentage of the total input and then normalized to each immunoglobulin G (IgG). The data are presented as the mean ± SEM, *n* = 3. ***p < 0.001. (**C**) ChIP analyses of putative AR response element 1 (ARE1) in the *pri-miR-203* promoter region in LNCaP cells following MDV3100 treatment for 24 h. The data are presented as the mean ± SEM, *n* = 3. *p < 0.05. (**D**) Activity of a red fluorescent protein (RFP) reporter gene containing the putative ARE1 from the *pri-miR-203* promoter. Expression of the transiently transfected reporter gene was assayed in LNCaP cells following co-transfection with a plasmid expressing AR, FOXA1, or an empty vector (EV). Relative multiples of the median fluorescence intensity (MFI) are given after normalization to a control vector. *vs. EV; ^#^−DHT vs. +DHT. The data are presented as the mean ± SEM, *n* = 3. *p < 0.05, **p < 0.01, ***p < 0.001. (**E**) MFIs of the *pri-miR-203* promoter-RFP reporter, putative ARE1 and ARE1 site-specific mutants assayed in LNCaP-AR cells treated with vehicle or DHT for 24 h. The data are presented as the mean ± SEM, *n* = 3. **p < 0.01.

AR is known to translocate from the plasma membrane to the nucleus and can bind specific AR-response elements (AREs) to activate target genes [33]. To investigate how AR signaling transcriptionally regulates miR-203 expression, we carefully examined at the putative promoter region of the primary miR transcript that encodes miR-203 (*pri-miR-203*) and identified three putative AREs and several AR cofactor (FOXA1 and OCT1) response elements within the promoter region (Figure 2A). We then performed ChIP assays to test whether dihydrotestosterone (DHT) directly mediates the binding of nuclear AR to *pri-miR-203*. Using AR, FOXA1, or OCT1 antibodies, AR and AR cofactor/*pri-miR-203* chromatin complexes were immunoprecipitated from nuclear extracts of LNCaP cells following DHT treatment, and qPCR was used to analyze the ARE region of *pri-miR-203*. Nuclear AR and AR cofactor-binding signals were significantly increased at ARE1 but not ARE2 or ARE3 after DHT treatment (Figure 2B). We also tested whether anti-androgen attenuates AR binding to the *pri-miR-203* promoter and found that AR inactivation by treatment with an AR antagonist, MDV3100, in LNCaP cells decreased binding of AR to ARE1 in the *pri-miR-203* promoter (Figure 2C). Moreover, the binding of nuclear AR to ARE1 was induced in RasB1 [14, 29, 34–39] metastatic cells harboring a wild-type AR-inducible expression vector (AE-TRE) in response to DHT following doxycycline induction (Supplementary Figure S2A).

In addition, we performed promoter reporter assays to examine whether ARE1 sites in the promoter region of *pri-miR-203* were functional. We used a construct in which ARE1 from the *pri-miR-203* promoter was incorporated into an RFP reporter. LNCaP cells were co-transfected with the reporter construct and AR/FOXA1 expression vectors and then treated with DHT for 24 h. Reporter activities were measured at 48 h after transfection, and RFP reporter activity was normalized to a control vector. The reporter assay demonstrated that ARE1 from *pri-miR-203* indeed significantly increased reporter activity in response to DHT and was further induced when upon co-transfection with AR/FOXA1 expression vectors (Figure 2D). Moreover, mutating ARE1 in the *pri-miR-203*/ARE1 reporter partially disrupted the ability of DHT to induce reporter activity in LNCaP-AR cells, suggesting that *pri-miR-203* is transcriptionally upregulated by AR binding at the identified ARE (Figure 2E). Furthermore, we tested nuclear AR binding to the promoter region of *pri-miR-203* in RasB1 metastatic cells. The induction of ARE1 reporter activity in response to DHT was observed in cells co-transfected with the reporter construct and the AR expression vector, and the greatest ability of AR to induce reporter activity was found following AR cofactor transfection (Supplementary Figure S2B). These data are consistent with a mechanism whereby nuclear AR activates miR-203 expression through a direct physical interaction with the promoter region of *pri-miR-203*.

### AR increases miR-203 and decreases SRC expression

**Figure 3:**
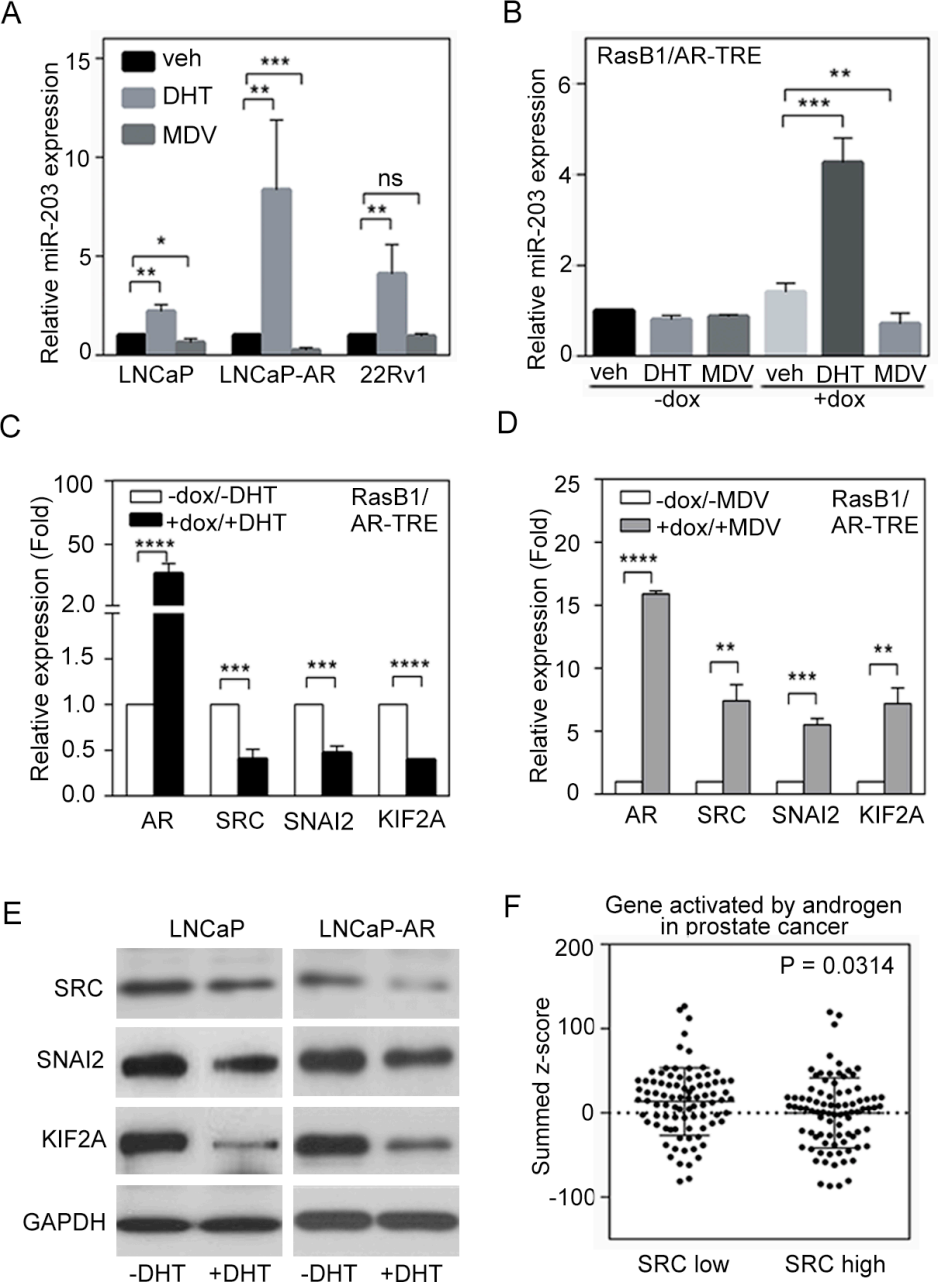
Androgen receptor (AR) signaling inversely regulates SRC and miR-203 levels (**A**) Relative miR-203 expression in LNCaP, LNCaP-AR, and 22Rv1 cells in the presence of vehicle, dihydrotestosterone (DHT), or MDV3100. The data are presented as the mean ± SEM, *n* = 3. *p < 0.05, **p < 0.01, ***p < 0.001. (**B**) Relative miR-203 expression in AR-TRE-transfected RasB1 cells with or without doxycycline (dox) induction in the presence of vehicle, DHT, or MDV3100. The data are presented as the mean ± SEM, *n* = 3. **p < 0.01, ***p < 0.001. (**C** and **D**) Relative AR, SRC, SNAI2, and KIF2A mRNA expression in AR-TRE-transfected RasB1 cells with or without doxycycline induction in the presence or absence of DHT (C) or MDV3100 (D). The data are presented as the mean ± SEM, *n* = 3. **p < 0.01, ***p < 0.001, ****p < 0.0001. (**E**) Relative SRC, SNAI2, and KIF2A protein expression in LNCaP and LNCaP-AR cells in the presence or absence of DHT. (**F**) Mean summed z-scores for AR signaling-responsive gene signatures in the Taylor PCa dataset (*n* = 111), showing that patients with high SRC expression had lower expression of genes that are activated by AR signaling. Statistical significance was determined by Student's *t*-test.

DHT treatment resulted in the induction of endogenous miR-203 levels in AR-positive LNCaP, LNCaP-AR (parental LNCaP overexpressing wild-type AR) [40], and CWR22Rv1 (22Rv1, a castration-resistant PCa cell line) cells, thereby establishing a positive correlation between AR and miR-203 in PCa cells, while the AR antagonist MDV3100 reduced relative miR-203 expression in LNCaP and LNCaP-AR cells but not in 22Rv1 cells (Figure 3A). Moreover, doxycycline-induced AR expression in RasB1/AR-TRE cells led to significantly increased miR-203 levels in cells with ligand activation, as demonstrated by DHT-mediated miR-203 increases, which were reversed with MDV3100 treatment in the presence of AR (Figure 3B). These data suggest that AR activates miR-203 expression in AR-positive PCa cells. Our previous work established that an AR-dependent transcriptional mechanism influences SRC activity [14]. In the current study, we hypothesized a molecular mechanism whereby a low canonical AR output contributes to reduced miR-203 and increased SRC levels. Investigating the molecular mechanisms involved in the relationship between AR pathway dysregulation and SRC expression in PCa cells, we found that SRC expression levels were decreased when LNCaP cells were treated with DHT, and increased SRC was observed in cells with MDV3100 treatment [14].

To further demonstrate that activated AR signaling reduces SRC expression in AR-negative cells, we introduced an AR expression vector into RasB1 cells. Following DHT treatment, we detected reduced SRC mRNA levels in these RasB1/AR-TRE cells (Figure 3C). Moreover, we showed that the mRNA levels of other known and predicted miR-203 targets, such as *SNAI2* and *KIF2A*, decreased in response to DHT following AR induction (Figure 3C) and increased in response to MDV3100 following AR induction (Figure 3D). We further confirmed that of the SRC, SNAI2, and KIF2A protein levels were reduced in LNCaP and LNCaP-AR cells upon DHT treatment (Figure 3E). These results support the idea that activated androgen-responsive signaling is associated with reduced SRC signaling in PCa.

To validate the mechanistic role of SRC in androgen-responsive signaling in PCa progression, we used a z-score analysis to examine the relationship between SRC expression and gene signatures that reflect activated AR signaling pathway components in the Taylor PCa dataset. Low levels of SRC expression were positively associated with high expression levels of AR signaling-activated genes in those samples (Figure 3F). Moreover, decreased levels of SRC expression were strongly associated with induced expression of AR pathway gene signatures [31] by GSEA in the Taylor PCa dataset, which is composed of gene expression data from the tissue specimens of 111 PCa patients, including 98 primary and 13 metastatic tumors (Supplementary Figure S3A). Similar results were obtained using a different database downloaded from the TCGA, including 50 early-stage prostate tumors (TCGA web site; Supplementary Figure S3B). However, we divided specimens from the Taylor PCa database into two groups with ‘low’ and ‘high’ AR or SRC expression, and a mean expression analysis showed that higher AR and SRC were significantly expressed in tissues with low levels of SRC and AR, respectively (Supplementary Figure S3C, S3D). We next analyzed the relationship between SRC and AR and found that AR and SRC expression was inversely correlated in the Taylor and TCGA databases (Supplementary Figure S3E, S3F). These data support an association between AR inactivation and SRC signaling activation in clinical PCa samples.

### miR-203 represses SRC signaling by directly binding to the *SRC* 3′UTR

**Figure 4:**
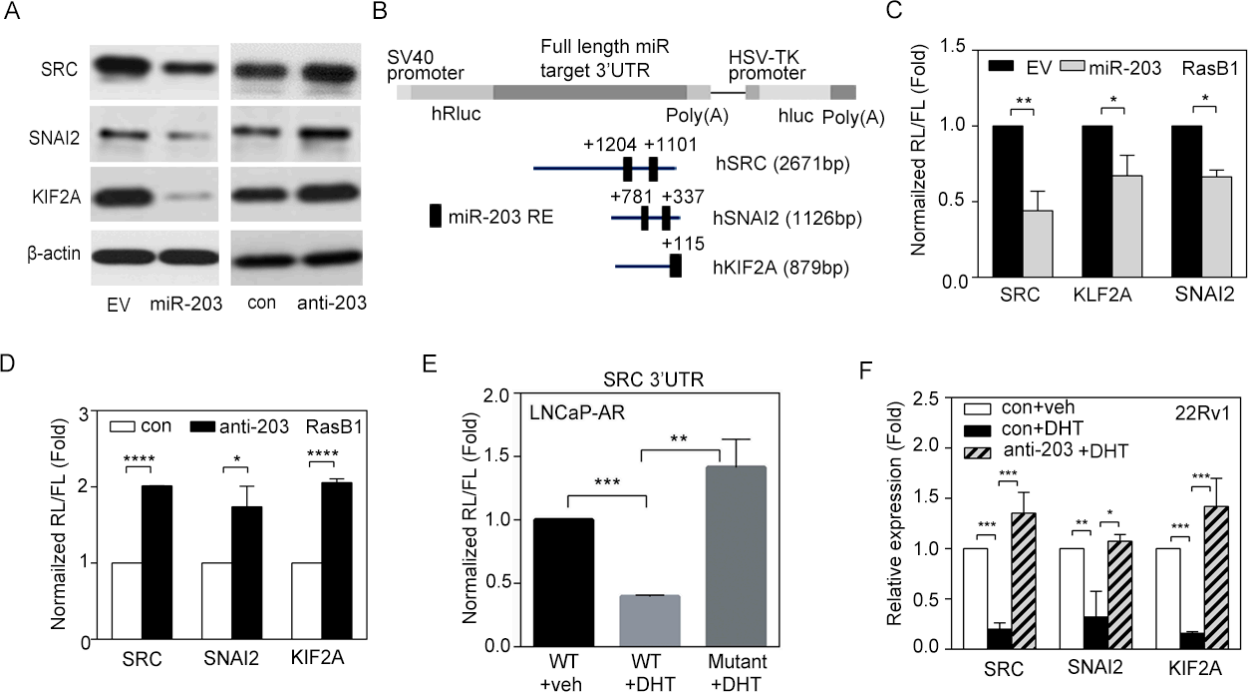
miR-203 mediates reductions in SRC, SNAI2, and KIF2A mRNA stability (**A**) SRC, SNAI2, and KIF2A protein expression in RasB1 cells transfected with empty vector (EV), miR-203 precursor, anti-miR control (con), or anti-miR-203 inhibitor. (**B**) Schematic of the predicted miR-203-response element (RE) in the 3′ untranslated region (UTR) of human *SRC, SNAI2*, and *KIF2A*. The full-length 3′UTRs from human *SRC, SNAI2*, and *KIF2A* were fused to the *Renilla* luciferase gene with the simian virus 40 (SV40) promoter in a bicistronic reporter construct that also expressed firefly luciferase from the herpes simplex virus thymidine kinase (HSV-TK) promoter. (**C**) Normalized 3′UTR reporter activities of *SRC, SNAI2*, and *KIF2A* in RasB1 cells transfected with EV or miR-203 precursor. *Renilla*/firefly luciferase (RL/FL) activities were measured 48 h after transfection. The data are presented as the mean ± SEM, *n* = 3. *p < 0.05, **p < 0.01. (**D**) Normalized 3′UTR reporter activities of *SRC, SNAI2*, and *KIF2A* in RasB1 cells transfected with anti-miR control or anti-miR-203 inhibitor. The data are presented as the mean ± SEM, *n* = 3. *p < 0.05, ****p < 0.0001. (**E**) Normalized reporter activity of the wild-type (WT) *SRC* 3′UTR (2671 bp) or a mutant containing modified miR-203-binding sites in LNCaP-AR cells treated with vehicle or DHT for 24 h. The data are presented as the mean ± SEM, *n* = 3. **p < 0.01, ***p < 0.001. (**F**) Relative SRC, SNAI2, and KIF2A mRNA levels in 22Rv1 cells treated with vehicle or DHT for 24 h after being transfected with anti-miR control or anti-miR-203 inhibitor. The data are presented as the mean ± SEM, *n* = 3. *p < 0.05, **p < 0.01, ***p < 0.001.

To examine the contribution of miR-203 and its potential targets to human PCa metastasis, we analyzed the specific role of miR-203 in repressing *SRC* gene expression in PCa. We found that ectopic expression miR-203 in the RasB1 metastatic PCa cell line was accompanied by decreases in the mRNA expression of SRC and other predicted miR-203 targets, SNAI2 and KIF2A (Supplementary Figure S4A). Moreover, when we expressed an anti-miR-203 inhibitor in RasB1 cells, we observed increases in SRC, SNAI2, and KIF2A mRNA levels (Supplementary Figure S4B). We further confirmed that SRC, SNAI2, and KIF2A protein expression was reduced and induced in RasB1 cells in response to miR-203 precursor and anti-miR-203 inhibitor, respectively (Figure 4A). These data suggest that miR-203 targets the *SRC, SNAI2*, and *KIF2A* genes, leading to decreases in SRC, SNAI2, and KIF2A expression at the post-transcriptional level.

Further exploring the molecular mechanism by which miR-203 affects SRC, SNAI2, and KIF2A expression in tumor cells, we identified predicted miR-203-binding sites in the 3′UTR regions of the SRC, SNAI2, and KIF2A mRNA transcripts (Figure 4B), suggesting that SRC, SNAI2, and KIF2A mRNAs are putative miR-203 targets. Three different reporter constructs containing full-length 3′UTRs from human SRC, SNAI2, and KIF2A mRNA were incorporated into a bicistronic luciferase reporter construct. RasB1 cells were co-transfected with the construct, and exogenous miR-203 precursor, *Renilla* and firefly luciferase activities were measured 48 h after transfection, with *Renilla* luciferase reporter activity normalized to the firefly luciferase control. The results showed that expression of the exogenous miR-203 precursor reduced reporter activity (Figure 4C). In addition, the expression of an anti-miR-203 inhibitor significantly increased the luciferase signal (Figure 4D). Moreover, we repeated the reporter assay using a reporter construct in which the predicted miR-203-binding sites in the 3′UTR region of *SRC* were mutated. The results showed that mutating the miR-203-binding sites in the 3′UTR region of *SRC* disrupted the ability of DHT to reduce reporter activity (Figure 4E), suggesting the sequence-specific downregulation of SRC by miR-203 upon AR activation. Importantly, we performed a miR-203 interruption assay to see if AR regulated SRC in 22Rv1 cells. Indeed, activating AR signaling by DHT treatment resulted in reduced levels of SRC and other miR-203 targets; however, pretreatment with anti-miR-203 disrupted this repressive effect (Figure 4F), confirming a novel mechanism in which activated AR signaling mediates the repression of SRC through the upregulation of miR-203. These data are consistent with the idea that miR-203 targets the 3′UTRs of *SRC, SNAI2*, and *KIF2A*, leading to the post-transcriptional inhibition of SRC, SNAI2, and KIF2A expression.

### AR-miR-203 and SRC levels are inversely correlated in human PCa

**Figure 5:**
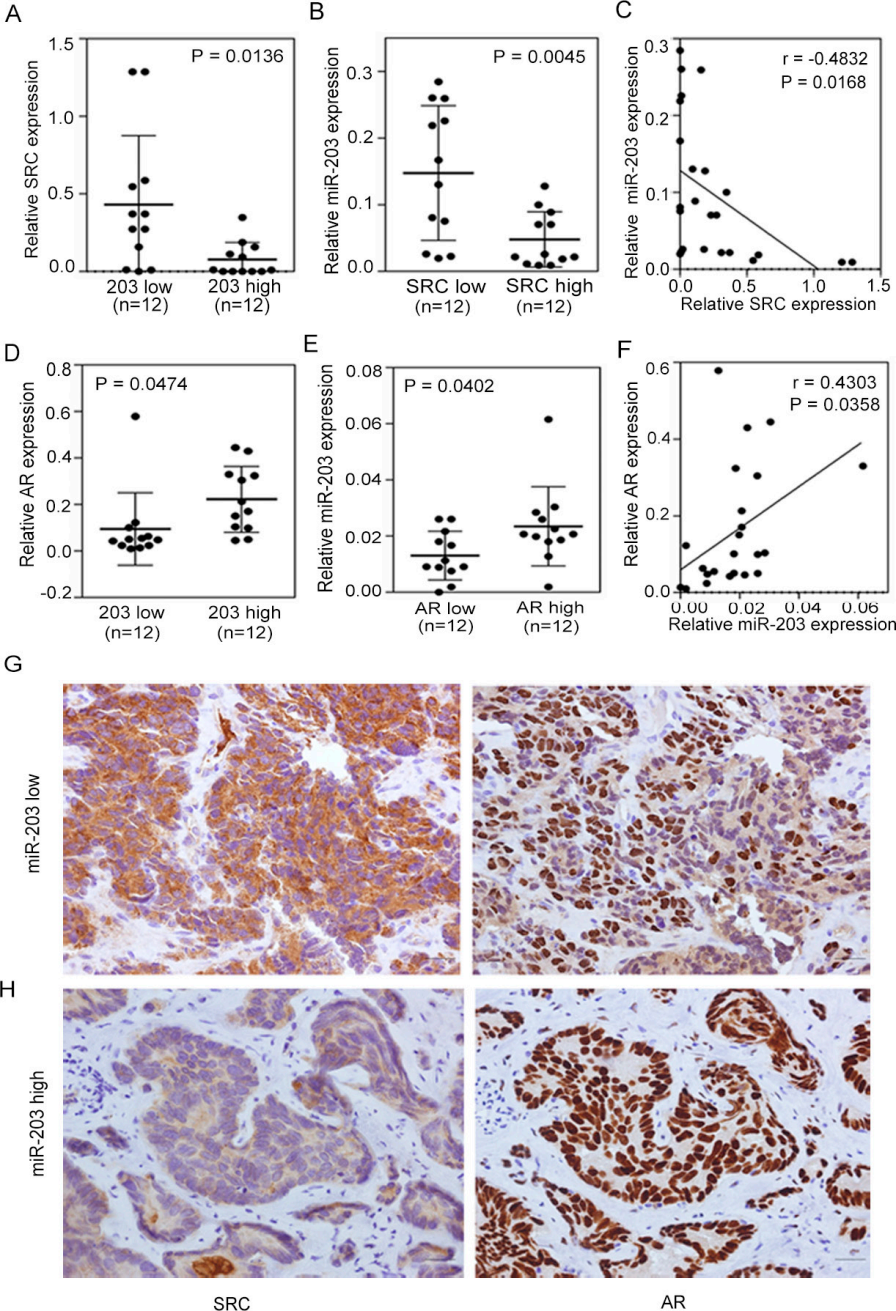
Induced SRC expression is associated with decreased androgen receptor (AR)-miR-203 expression in PCa (**A** and **B**) Patients with low miR-203 (A) or SRC (B) expression had higher SRC or miR-203 expression (*n* = 12 in each group). Significance was determined by a two-tailed test. (**C**) Inverse correlation between relative miR-203 expression and relative SRC mRNA expression in PCa samples (*n* = 24). Significance was determined by the Gaussian population (Pearson) test. (**D** and **E**) Patients with high miR-203 (D) or AR (E) expression had higher AR or miR-203 expression (*n* = 12 in each group). Significance was determined by a two-tailed test. (**F**) Positive correlation of relative AR expression to relative miR-203 expression in PCa samples (*n* = 24). Significance was determined by the Gaussian population (Pearson) test. (**G** and **H**) Immunohistochemical (IHC) staining of PCa tissue sections with low miR-203 (G) and high miR-203 (H) levels with antibodies specific for SRC (left panel) and AR (right panel). Scale bars represent 100 μm.

To further confirm the role of AR and establish its relationship with miR-203 and SRC, we directly monitored expression levels in tissue samples from PCa patients. First, we analyzed mRNA levels in 24 independent prostate tumors collected from Wan Fang Hospital, Taipei Medical University (Taiwan). Samples were divided into two groups based on miR-203 and SRC expression levels, and an ANOVA showed that tissues with higher miR-203 levels had lower SRC levels (Figure 5A) and that lower SRC levels were correlated with higher miR-203 expression levels (Figure 5B). In addition, a significant negative correlation between SRC and miR-203 levels was confirmed by statistical analyses of clinical samples (Figure 5C). We further investigated the correlation between AR and miR-203 levels and observed higher miR-203 expression in tumors that had higher AR expression, and vice versa (Figure 5D, 5E). Moreover, a significant positive correlation was found between AR and miR-203 according to a Pearson coefficient correlation analysis (Figure 5F).

We also performed Immunohistochemical (IHC) staining to examine SRC and AR protein levels in these 24 PCa samples based on the levels of miR-203. Consistent with our finding that AR and SRC expression is miR-203-dependent, we observed strong SRC expression in PCa samples with lower miR-203 levels (Figure 5G, left) compared with tissue samples with higher miR-203 expression, which showed reduced SRC staining (Figure 5H, left). However, when we monitored AR levels in continuous tissue sections, tumors with lower miR-203 levels showed reduced AR staining (Figure 5G, right) compared with tissue samples with high miR-203 expression, which showed elevated AR staining (Figure 5H, right). These results are consistent with our proposed mechanism that SRC expression is dependent on miR-203 expression status via AR activation.

### SRC expression reconstitutes cell growth and motility in miR-203-expressing cell

**Figure 6:**
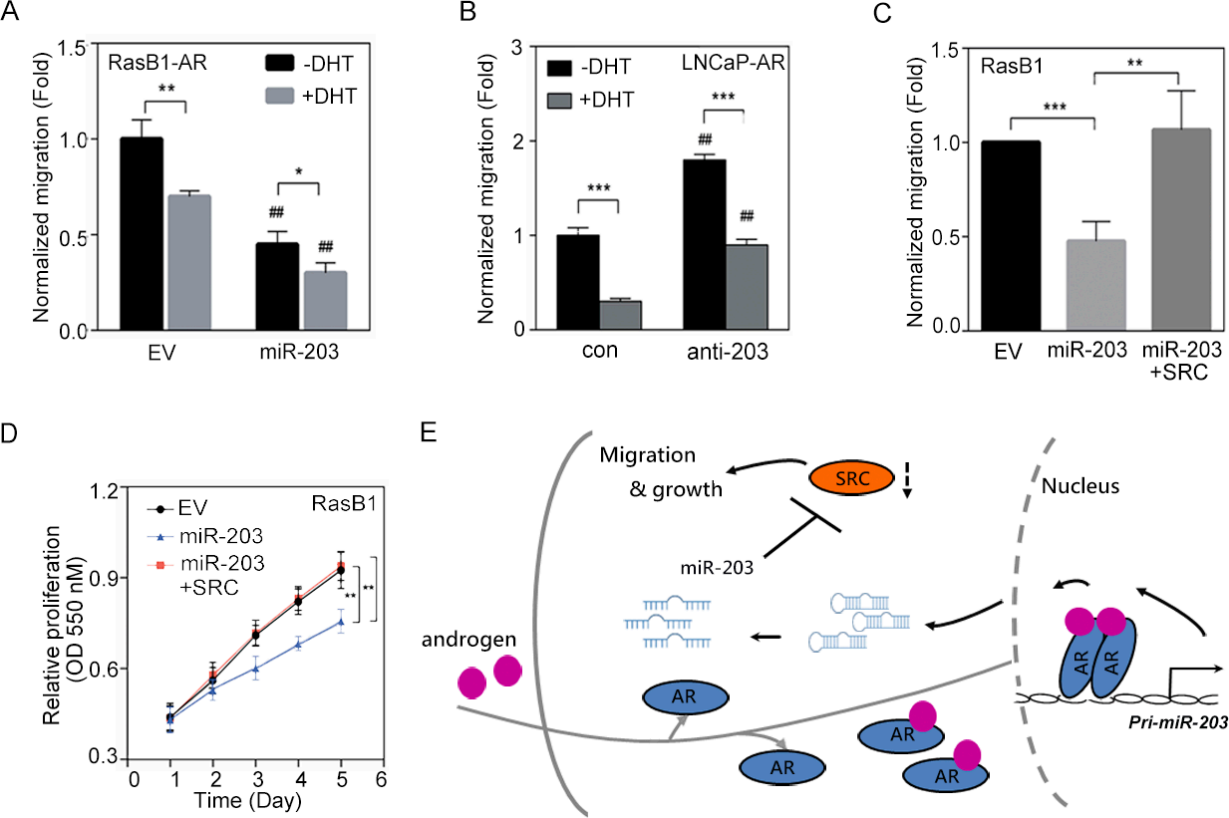
SRC expression reconstitutes the malignant phenotype in miR-203-expressing PCa cells (**A**) Normalized migration of RasB1-AR cells transfected with empty vector (EV) or miR-203 precursor in the presence of dihydrotestosterone (DHT) for 24 h. Cells that had migrated through the filter were quantified by an ELISA reader at 550 nm in triplicate. ^#^ EV vs. miR-203. The data are presented as the mean ± SEM, *n* = 3. *p < 0.05, **p < 0.01. (**B**) Normalized migration of LNCaP-AR cells transfected with anti-miR control (con) or anti-miR-203 inhibitor in the presence of DHT. ^#^ con vs. anti-miR-203. The data are presented as the mean ± SEM, *n* = 3. **p < 0.01, ***p < 0.001. (**C**) Normalized migration of RasB1 cells with EV, miR-203 expression, or miR-203 reconstituted with SRC. The data are presented as the mean ± SEM, *n* = 3. **p < 0.01, ***p < 0.001. (**D**) Proliferation of RasB1 cells transfected with EV, miR-203, or miR-203 reconstituted with SRC. The data are presented as the mean ± SEM, *n* = 5. **p < 0.01. (**E**) Proposed model for androgen stimulation of nuclear AR-dependent *pri-miR-203* transcription and pre-miR-203 being processed to miR-203. miR-203 inhibits the translation of SRC transcripts by binding their 3′ untranslated region (UTR) and thus inactivates the cell-migration and growth effects of SRC expression.

SRC signaling plays critical roles in the development of PCa malignancy [14, 41]. Our results showed that AR activation increased miR-203 expression and consequently suppressed SRC expression (Figure 3); we further examined the functional relevance of AR-increased miR-203 in reducing the motility of PCa cells. As shown in Figure 6A, when we treated RasB1 metastatic cells that harbored a constitutive AR expression vector (RasB1-AR) with DHT, the migration of these cells decreased, according to transwell assays, and a further decrease in migration was found following miR-203 precursor transfection. To assess the contribution of androgen-regulated miR-203 to cell motility, we transiently introduced an anti-miR-203 inhibitor or a control inhibitor into LNCaP-AR cells and treated the cells with DHT. Transfection with anti-miR-203 in LNCaP-AR cells induced cell motility, as observed in migration assays, and reduced cell motility was found when cells were treated with DHT (Figure 6B). Importantly, several studies showed that physiological concentrations of DHT can suppress the proliferation of AR-positive cell lines derived from PCa and the ectopic expression of AR [42–44]. We recapitulated the inhibitory effect of DHT using a proliferation assay in an LNCaP-derived cell line and found that DHT-treated cells exhibited reduced cell proliferation (Supplementary Figure S5A). Moreover, anti-miR-203-expressing cells displayed significantly induced growth rates in response to DHT (Supplementary Figure S5A), suggesting that miR-203 inhibits the proliferation of AR-positive cells in a manner related to AR activation. Having demonstrated that the ectopic expression of a miR-203 precursor decreased SRC expression (Figure 4), we then asked whether the reconstitution of SRC levels in miR-203-expressing cells was sufficient to restore cell motility. RasB1 metastatic cells with ectopic miR-203 expression had reduced migration in transwell assays compared with cells that carried the empty vector (Figure 6C). We transfected these cells with a SRC expression vector, and as expected, the induction of SRC expression increased cell motility even in the presence of high miR-203 expression (Figure 6C), implying that SRC acts downstream of miR-203. We further examined the functional relevance of the SRC-mediated induction of proliferation in miR-203-expressing RasB1 and PC3 metastatic cells. Rescuing SRC expression in RasB1 and PC3 cells harboring miR-203 precursor significantly induced cell growth (Figure 6D and Supplementary Figure S5B). These results support *SRC* as a miR-203 target for determining cell growth and motility. Taken together, our data demonstrate that *pri-miR-203* transcription is directly and positively modulated by AR, resulting in the inactivation of SRC-stimulated malignant phenotypes (Figure 6E).

## DISCUSSION

PCa remains the main cause of cancer deaths in men worldwide, as conventional strategies for treating PCa are still unsatisfactory. The challenge of understanding this dynamic process resides in unraveling regulatory networks involving transcription factors and miRs. Here, we investigated miRs regulated by the AR and their potential roles in regulatory networks underlying prostate malignancy. To our knowledge, our study is the first to reveal that miR-203 is transcriptionally regulated and activated by AR or DHT treatment in PCa cells. We also found that miR-203 decreased SRC expression in PCa, which in turn inhibited cell growth and migration. By combining computational biology and experimental approaches, we propose a novel network integrating AR, miR-203, and SRC.

Crosstalk between AR and SRC has been observed in human cancer [9–11]. SRC was reported to mediate the phosphorylation of AR, which is associated with nuclear translocation and the activation of AR-responsive transcription [45, 46]. Reciprocally, SRC activity can be increased non-genomically by the cytoplasmic AR independent of its transcriptional activity [47–49]. Interestingly, previous transcriptomic analyses showed that SRC-dependent transcription is inversely correlated with canonical AR outcomes following treatment with an SRC inhibitor (i.e., dasatinib) [50]. Importantly, in clinical samples, increased SRC activity and an SRC-dependent transcription signature were shown to be correlated with decreased AR output [13]. In our study, we propose the negative regulation of SRC by AR through miR-203, whereby a low canonical AR output contributes to increased SRC and reduced miR-203 levels.

miRs are known to be targets of transcription factors [27, 51]. We show that miR-203 can be activated by AR and subsequently suppress cancer progression. However, miR-203 was shown to be repressed by the transcriptional repressor *SNAI1/2*, forming a double-negative miR-203/SNAI1/2 feedback loop in breast cancer [52]. Consistent with our previous report, SNAI1 directly binds to the *pri-miR-203* stem-loop promoter and functions to inhibit miR-203 transcription in PCa [29]. Our present results suggest that miR-203 serves as a tumor suppressor, whereby AR activates miR-203, and the induction of miR-203 reduces SNAI2 levels. Importantly, individual miRs are capable of regulating dozens of distinct mRNAs [53–55]. We considered the possibility that either other miRs are involved or that miR-203 might act on several other target genes besides SRC in response to AR signaling. Although miR-203 was not reported to be regulated by androgen with global profiling studies of microRNAs [56, 57], we found a significant positive correlation between AR and miR-203 in clinical samples and identified AR as a direct regulator of miR-203 expression. These results present a new mechanism underlying the malignant phenotype in PCa treatment, in which androgen deprivation downregulates miR-203 levels, which in turn results in SRC overexpression. Therefore, miR-203 can be used as a potential prognostic marker and therapeutic target in human PCa.

Studies with the new anti-androgen agent MDV3100 (enzalutamide) indicate that one of the ways it inactivates AR nuclear activity in tumor cells is by preventing nuclear translocation [58, 59]. However, non-genomic signaling of AR can contribute to SRC activation [60–62]. Activated SRC was shown to be correlated with resistance to enzalutamide in castration-resistant patients [63]. One concern is that overlapping mechanisms might result in increased castration-resistance caused by SRC activation. Alternatively, we revealed that the aberrant downregulation of miR-203 is partially responsible for increased SRC expression and subsequent activation in PCa. Our study demonstrated that targeting SRC using a miR-203 mimic would be a promising therapeutic strategy for treating SRC signaling-activated PCa patients.

## MATERIALS AND METHODS

### Clinical outcomes and correlation analyses using human gene expression datasets

To compare miR-203 expression levels with PCa progression and survival and with AR and SRC expression levels, we used miR and mRNA expression data from the Taylor PCa dataset [30] and the Cancer Genome Atlas (TCGA) PCa dataset. The study using the Taylor dataset was accessed from the Memorial Sloan Kettering Cancer Center (MSKCC) Cancer Genomics data portal (http://cbio.mskcc.org/cancergenomics/prostate/data/) on 7/27/2012, and clinical and publicly available gene expression and microRNA expression data on 98 primary and 13 metastatic PCa samples were downloaded. The study using TCGA dataset was selected Level 3 normalized microarray gene expression data (UNC_AgilentG4502A_07) from TCGA database (https://tcga-data.nci.nih.gov/tcga/; 11/21/2014) of 372 primary and 50 early-stage prostate tumors from patients treated by a radical prostatectomy. Expression data (and resulting z-scores) were log2-normalized. For the gene set enrichment analysis (GSEA), GSEA software was downloaded from the Broad Institute [64], and gene sets of AR-dependent [31, 32] responsive gene signatures were used to determine correlations with miR-203 and SRC levels. Total human PCa samples (containing expression data from 98 primary and 13 metastatic prostate tumors) from the MSKCC and 50 early-stage prostate tumors from TCGA were assigned to two groups based on the medians of miR-203 and SRC expressions. The number of permutations was set to 1000, and the permutation type was set to “phenotype”. A normalized enrichment score (NES) and false discover rate (FDR) were calculated by the program. Correlations of miR-203 and SRC with the gene set were suggested by NES values. For the z-score analysis, gene sets were scored by summing the expression z-scores per tumor within the cohort. Tumors were mean-stratified by miR-203, SRC, and AR expressions, and the mean expression of each of these genes was determined in each group.

### Cells, constructs, and reagents

LNCaP, PC3, and 22Rv1 human PCa cell lines were obtained from ATCC (MD, USA). The LNCaP-AR and metastatic RasB1 cell lines were provided by Dr. Kathleen Kelly (NCI/NIH, MD, USA). RasB1 cells metastasize to bone with a high frequency and were maintained as described previously [14, 29, 34–39]. All cells were authenticated within 6 mon prior to use by a morphology check and growth curve analysis according to the provider's recommendations. All PCa cell lines were cultured in RPMI 1640 medium supplemented with 10% fetal bovine serum (FBS). RasB1 cells with inducible AR expression (AR-TRE) were subcloned into the pFUGW lentiviral vector with a TRE3G promoter and an IRES-mcherry reporter. Cells with stable or transient expression of AR, SRC, FOXA1, OCT1, or miR-203 were established by transfection with either an AR, SRC, FOXA1, or OCT1 expression vector, miR-203 precursor, or empty vector pCDH-CMV-MCS-EF1-Puro (System Biosciences, CA, USA) with a puromycin-selectable marker. Transient transfections of plasmids and anti-miRs inhibitors were carried out using the X-tremeGENE HP DNA transfection reagent (Roche, CA, USA) or Lipofectamine RNAiMAX (Invitrogen, CA, USA), respectively. Some cells were treated with the AR antagonist, MDV3100, at 10 μM for 24 h in 10% FBS-containing medium. Dihydrotestosterone (DHT) treatment (10 nM) was carried out in 10% charcoal-stripped FBS-containing medium (Invitrogen, CA, USA). DHT was from Sigma-Aldrich (MO, USA), and MDV3100 was from Selleck (TX, USA). Anti-miR inhibitors (control and anti-miR-203) were from GeneCopoeia (MD, USA). The AR response elements (putative AREs: ARE1, ARE2, and ARE3) were respectively located upstream of human *pri-miR-203* on chromosomes 14:104582145, 14:104582449, and 14:104582665 at GRCh37. The *pri-miR-203* promoter with ARE-red fluorescent protein (RFP) reporter vectors was constructed using the Clone-it Enzyme free Lentivectors Kit (System Biosciences, CA, USA). Human *SRC, SNAI2*, and *KIF2A* with miR-203 response element 3′UTR reporters were constructed using the psiHECKTM-2 vector (Promega, WI, USA). The miR-203-binding sites of *SRC* 3′UTR and putative ARE1 of *pri-miR-203* promoter-RFP mutations were made using a Site-Directed Mutagenesis System kit (Invitrogen, CA, USA). All primers used for these constructs are listed in Supplementary Table S1. All constructs were verified by a DNA sequence analysis.

### Chromatin immunoprecipitation (ChIP) assay

ChIP assays were performed using the EZ magna ChIP A kit (Millipore, CA, USA) with a modified protocol. For each sample, 10^7^ cells in 10-cm dishes were treated with DHT (10 nM) as indicated in 10% charcoal-stripped FBS-containing medium for 24 h. Cultured cells were cross-linked with 1% formaldehyde in culture medium at room temperature for 15 min. Fixation was quenched by the addition of 1 ml of 10× glycine, and cells were washed twice with cold phosphate-buffered saline (PBS) containing a complete protease inhibitor (Roche, CA, USA). Harvested cells were centrifuged at 10^4^ rpm, and cell pellets were resuspended in 0.5 ml of cell lysis buffer containing 1× protease inhibitor and incubated on ice for 15 min. Nuclei were collected by centrifugation at 10^4^ rpm and 4°C for 10 min and resuspended in nuclear lysis buffer. Chromatin was sheared using a sonicator (Branson Sonifier 250, Germany) with a microtip in a 20-second burst followed by 1 min of cooling on ice for a total sonication time of 5 min/sample. This procedure resulted in DNA fragments of approximately 100–300 bp. Sheared chromatin was divided to perform immunoprecipitation with a rabbit immunoglobulin G (IgG) antibody or a primary antibody at 4°C overnight. Immunoprecipitation, washing, elution, reverse crosslinking, and DNA-purification steps were performed according to Millipore's protocol. A quantitative polymerase chain reaction (qPCR) was performed in triplicate with 1 μl of eluted chromatin. ChIP antibodies and PCR primers are listed in Supplementary Table S2. Predictions for putative ARE, FOXA1, and OCT1 transcription factor-binding sites within promoter regions were adopted from the AliBaba 2.1 program (gene-regulation.com).

### Promoter reporter assay

Promoter function was analyzed using fluorescence-activated cell sorting (FACS), and relative median fluorescent intensity (MFI) values were measured as previously described [14, 29, 34, 35, 39]. The MFI value for RFP was measured by FACS using FACSDiva software and was normalized to the value of the vehicle. LNCaP and RasB1 cells transfected with 1 μg of *pri-miR-203* promoter reporter were transiently co-transfected with 1 μg of AR and/or a FOXA1 or OCT1 expression vector or empty vector in the presence or absence of 10 nM DHT in 10% charcoal-stripped FBS-containing medium.

### Real-time reverse-transcription (RT)-PCR

An RT-PCR was used to measure miR-203, AR, SRC, SNAI2, and KIF2A expressions in LNCaP, LNCaP-AR, and 22Rv1 cell lines in the presence and absence of DHT or MDV3100 stimulation and/or RasB1 cells with AR-TRE transfection in the presence and absence of doxycycline, DHT, or MDV3100. Total RNA was isolated using the mirVana PARIS RNA isolation system (Ambion, TX, USA). For mRNA RT, 3 μg of total RNA was used with the SuperScript III kit (Invitrogen, CA, USA). The amplification step used the SYBR green PCR master mix (Applied Biosystems, MA, USA). For all mRNA primer pairs, the thermocycler was run for an initial 95°C incubation for 10 min, followed by 40 cycles with 95°C for 15 seconds and 60°C for 1 min. All reactions were normalized to human *GAPDH* expression and run in triplicate. All primers used for the PCR are listed in Supplementary Table S3. miR RT-PCRs were performed using a TaqMan MicroRNA Assay kit (Applied Biosystems, MA, USA). All values were normalized to the human *SNORD48* endogenous control and run in triplicate. miR-203, AR, and SRC levels in clinical samples used in qRT-PCR analyses were collected from 24 patients with independent prostate tumors at Wan Fang Hospital, Taipei Medical University (Taiwan). RNA was extracted from dissected tissue containing > 70% tumor cell content. The method for determining the specimens into two groups of ‘low’ and ‘high’ miR-203, AR, and SRC expressions was pre-decided by half of the number of patients according to miR-203, AR, or SRC levels by a qRT-PCR.

### Western blot analysis

Cells grown on 6-well plates (10^6^ cells/well) were lysed in 150 μl RIPA buffer containing complete protease inhibitors (Roche, CA, USA) and phosphatase inhibitors (Roche, CA, USA), 25 mM β-glycerophosphate, 10 mM sodium fluoride, and 1 mM sodium vanadate. Twenty micrograms of protein was separated per lane by sodium dodecylsulfate (SDS)-gel electrophoresis. After transfer to polyvinylidene difluoride membranes, blots were blocked with 5% bovine serum albumin (BSA) in PBST (PBS and Tween 20). Primary antibodies were incubated overnight at 4°C, and secondary antibodies were incubated at room temperature for 1 h as indicated in Supplementary Table S4.

### 3′UTR luciferase assay

For the miR target reporter assays, RasB1 cells in 12-well plates (5 × 10^4^ cells/well) were transiently transfected with 1 μg of the *SRC*, *SNAI2*, and *KIF2A* 3′UTR reporters and 1 μg of the miR-203 precursor or 50 nM of the anti-miR-203 inhibitor. Cell extracts were prepared in PBS 48 h after transfection, and luciferase activities were measured using the Dual Luciferase Reporter Assay System (Promega, WI, USA). Three independent experiments were run with triplicate samples. miR-binding sites were identified using the Computational Biology Center, MSKCC (microRNA.org), and Bioinformatics and Research Computing, Whitehead Institute for Biomedical Research websites (TargetScan. org).

### Immunohistochemical (IHC) staining

Twenty-four cases of independent primary prostatic adenocarcinomas were collected from Taipei Medical University-Wan Fang Hospital (Taiwan). The study was approved by the Taipei Medical University-Wan Fang Hospital Institutional Review Board (approval no.: N201512066) and carried out in accordance with the approved guidelines. IHC staining of SRC and AR proteins and their localization were performed using a rabbit monoclonal-SRC (Cell Signaling Technology, MA, USA) and a rabbit monoclonal-AR (Epitomics, CA, USA) antibodies at 1:6000 (SRC) and 1:250 (AR) dilution. In general, unstained sections were deparaffinized and rehydrated. Antigen retrieval was performed using the Target Antigen Retrieval Solution (DAKO, CA, USA), and autoclaved for 10 min. Endogenous peroxidase was blocked using a 3% hydrogen peroxide solution. All sections were blocked with the Cyto Q Background Buster Reagent (Innovex BioSciences, CA, USA). Primary antibodies were incubated overnight at 4°C in antibody diluent with background reducing components (DAKO, CA, USA). The secondary antibody, 1:250 horseradish peroxidase-labeled anti-mouse/rabbit (Vector Laboratories, CA, USA), was incubated at room temperature for 30 min, and bound peroxidase was detected using the ABC Peroxidase Kit (Vector Laboratories, CA, USA) and DAB (DAKO, CA, USA). All IHC slides were counterstained with hematoxylin. For a histomorphometric analysis of tissue sections, microscopic images were examined under 400× magnification using an Axioplan microscope system (Zeiss, NY, USA).

### Migration assay

RasB1-AR and LNCaP-AR cells were transiently transfected with the miR-203 precursor or an empty vector and/or an anti-miR-203 inhibitor or a control inhibitor and treated with 10 nM DHT in 10% charcoal-stripped FBS-containing medium. RasB1 and PC3 cells were transiently transfected with miR-203 precursor and/or SRC expression vector. In total, 2.5 × 10^5^ cells/well in serum-free medium were plated in the upper chamber of a transwell plate (BD Falcon, NJ, USA). The lower chamber was filled with 600 μl of serum-containing medium. Cells that had migrated through the transwells after 24 h were fixed and stained with a 0.5% crystal violet fixative solution for 15 min. Migrated cells on the underside of the membrane were counted and quantified by an enzyme-linked immunosorbent assay (ELISA) reader at 550 nm in triplicate.

### Proliferation assay

LNCaP-AR cells were transiently transfected with an anti-miR-203 inhibitor or a control inhibitor and treated with 10 nM DHT in 10% charcoal-stripped FBS-containing medium. RasB1 and PC3 cells were transiently transfected with miR-203 precursor and/or SRC expression vector. Cells were seeded at a density of 2 × 10^3^ cells/well in 96-well plates. The experiment was performed with multiple wells at each time point and then averaged. Each day, one plate was stained with a 0.5% crystal violet fixative solution for 15 min, rinsed with distilled water, and allowed to air-dry. At the end of the experiment, the crystal violet was dissolved by adding 100 μl of 50% ethanol containing 0.1 M sodium citrate to each well, and the absorbance was quantified at 550 nm on a plate reader.

### Statistical analysis

All data are presented as the mean ± standard error of the mean (SEM). Statistical calculations were performed with GraphPad Prism analytical tools. Differences between individual groups were determined by Student's *t*-test or a one-way analysis of variance (ANOVA) followed by Bonferroni's post-test for comparisons among three or more groups. The method for determining cutoff points was pre-decided by taking half the number of patients. *p* values of <0.05 were considered to be statistically significant.

## SUPPLEMENTARY MATERIALS FIGURES AND TABLES


