# Supplementary material for: Androgen receptor regulates SRC expression through microRNA-203
Source: Oncotarget. 2016 Mar 25;7(18):25726–41. doi: 10.18632/oncotarget.8366 (PMC5041939; doi:10.18632/oncotarget.8366)
Supplement: Supplementary file 1 [file oncotarget-07-25726-s001.pdf]

Androgen receptor regulates SRC expression through microRNA-203

Supplementary Materials

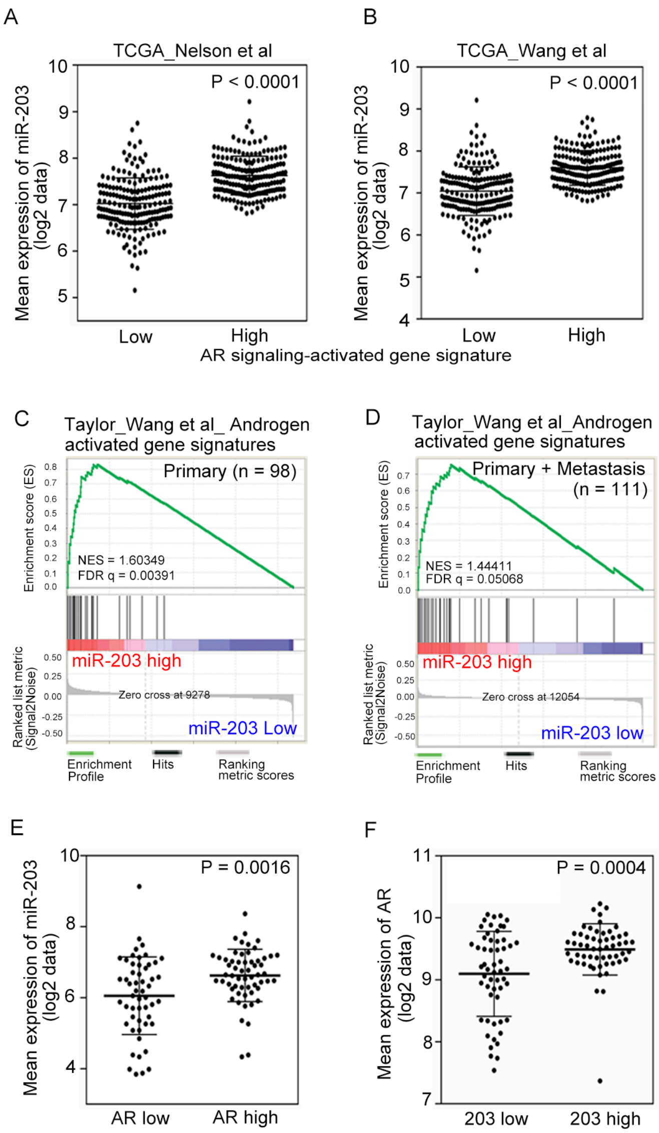

Supplementary Figure S1: miR-203 expression is associated with androgen-activated gene signatures. (**A** and **B**) Mean expression of miR-203 in the TCGA PCa dataset (n = 372) relative to gene sets from Nelson et al. (A) and Wang et al. (B), the expression of which increased or decreased with AR signaling in PCa tissues. Statistical significance was determined by Student's t-test. (**C** and **D**) Gene set enrichment analysis (GSEA) of the Taylor PCa dataset of primary (n = 98) (C) and combined primary and metastatic (n = 111) (D) tumors showing enrichment of miR-203 expression in the gene set of Wang et al., the expression of which was associated with patients with increased AR signaling. NES, normalized enrichment score; FDR, false discovery rate. (**E** and **F**) Mean expression of miR-203 (E) and AR (F) in the Taylor PCa dataset (n = 111), showing that patients with high AR and miR-203 expression had higher expression of miR-203 and AR, respectively. Statistical significance was determined by Student's t-test.

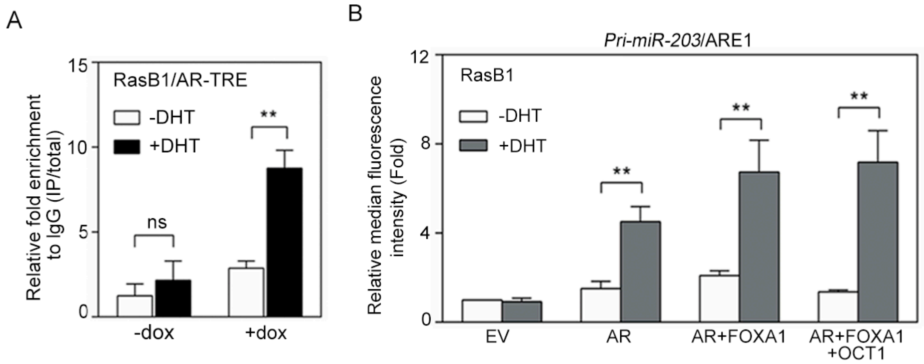

Supplementary Figure S2: AR directly and positively binds to the *pri-miR-203* promoter. (**A**) Chromatin immunoprecipitation (ChIP) analyses of putative ARE1 in the *pri-miR-203* promoter region in RasB1/AR-TRE cells following treatment for 24 h with DHT or doxycycline (dox). The data are presented as the mean ± SEM, $n = 3$. **$p < 0.01$. (**B**) Median fluorescence intensity (MFI) of the *pri-miR-203* promoter-RFP reporter, putative ARE1 assayed in RasB1 cells with a plasmid expressing empty vector (EV), AR, FOXA1, or OCT1 following treatment for 24 h with DHT. The data are presented as the mean ± SEM, $n = 3$. **$p < 0.01$.

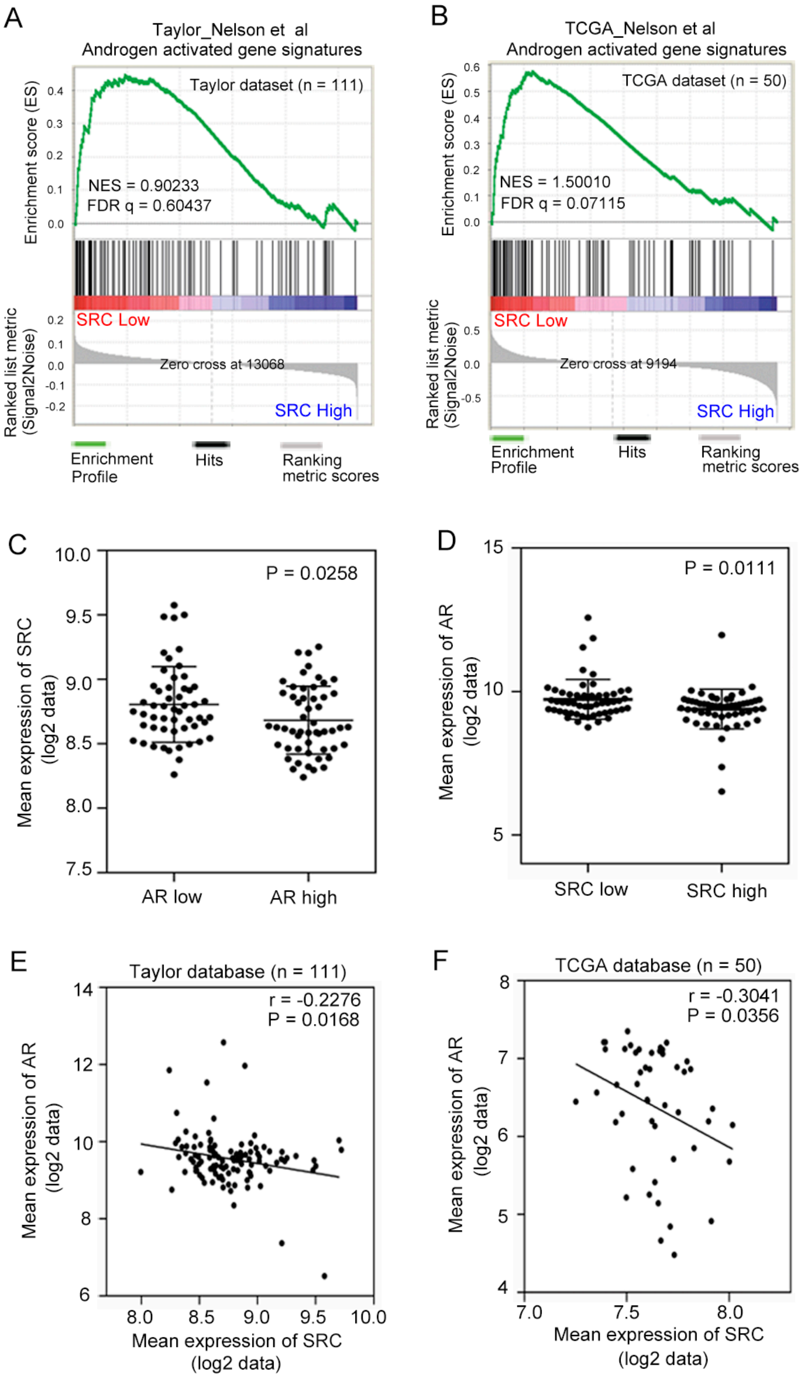

Supplementary Figure S3: SRC was inversely correlated with androgen receptor (AR) signaling profile and AR level. (**A** and **B**) Gene set enrichment analysis (GSEA) of the Taylor PCa dataset (n = 111) (A) and the TCGA dataset (n = 50) (B) showing enrichment of AR signaling-responsive gene signatures among the gene set of Nelson et al., the expression of which was associated with patients with decreased SRC expression. NES, normalized enrichment score; FDR, false discovery rate. (**C** and **D**) Patients with low AR expression had high SRC expression (C), and patients with low SRC expression had high AR expression (D), in the Taylor PCa dataset (n = 111). Statistical significance was determined by Student's t-test. (**E** and **F**) Pearson coefficient correlation analysis of the mean AR to mean SRC expression in the Taylor dataset (n = 111) (E) and the TCGA dataset (n = 50) (F). Significance was determined by the Gaussian population (Pearson) test.

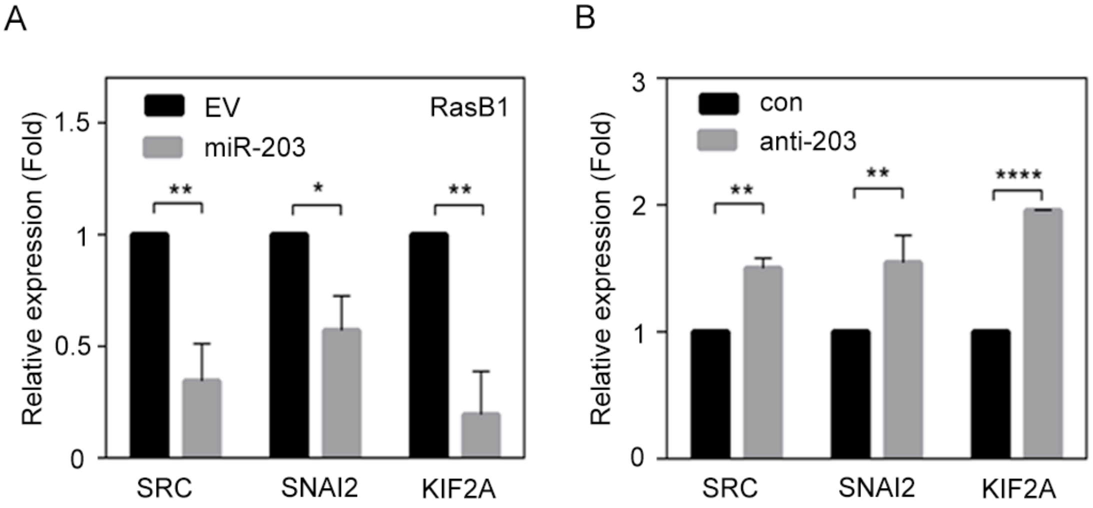

Supplementary Figure S4: miR-203 reduces SRC, SNAI2, and KIF2A expression. (**A**) Relative SRC, SNAI2, and KIF2A mRNA levels in RasB1 cells transfected with empty vector (EV) or miR-203 precursor. The data are presented as the mean ± SEM, $n = 3$. *$p < 0.05$, **$p < 0.01$. (**B**) Relative SRC, SNAI2, and KIF2A mRNA levels in RasB1 cells transfected with anti-miR control (con) or anti-miR-203 inhibitor. The data are presented as the mean ± SEM, $n = 3$. **$p < 0.01$, ****$p < 0.0001$.

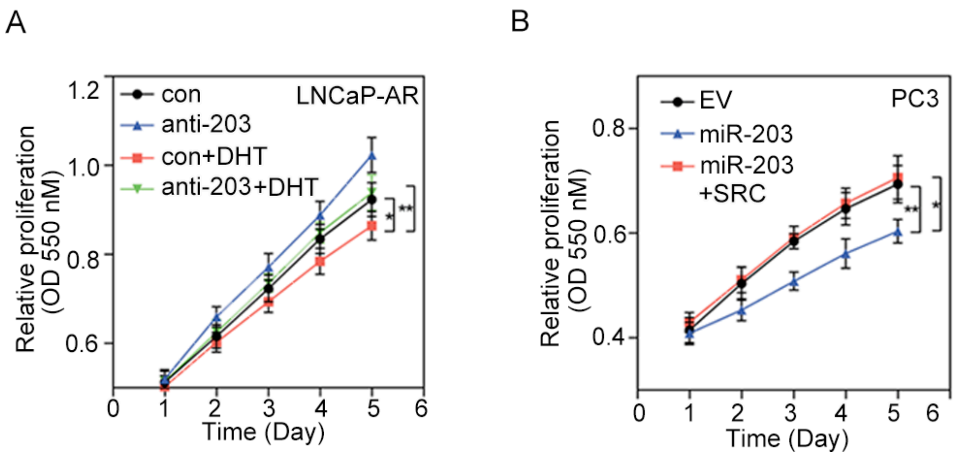

Supplementary Figure S5: miR-203 inhibits the proliferation of AR-positive cells and rescuing SRC expression reconstituted cell growth. (**A**) Proliferation of LNCaP-AR cells transfected with anti-miR control (con) or anti-miR-203 inhibitor in the presence of DHT. The data are presented as the mean ± SEM, $n = 6$. $^{*}p < 0.05$, $^{**}p < 0.01$. (**B**) Proliferation of PC3 cells transfected with empty vector (EV), miR-203, or miR-203 reconstituted with SRC. The data are presented as the mean ± SEM, $n = 5$. $^{*}p < 0.05$, $^{**}p < 0.01$.

Supplementary Tables

Supplementary Table S1: Primer sequences of the 3′ untranslated region (UTR) and promoter reporter constructs

**Primary hsa-miR-203 promoter reporter constructs**	
Pri-mir-203 P1	CAAACCCTCGTGGGATGTAG
Pri-mir-203 P2	CGTACCGGACGCCTTAGAC
Pri-mir-203 P3	gaggcagcagagaccgCAAACCCTCGTGGGATGTAG
Pri-mir-203 P4	cgaacagagagagaccgCGTACCGGACGCCTTAGAC
Pri-mir-203 ARE1M F	GAGCTCCCAAGCTCCGGATCCTGATCTCAAG
Pri-mir-203 ARE1M R	GGAGCTTGGGAGCTCCTCCCCCAACACAA
**3′UTR reporter construct primer sequences**	
SRC/psi-2 3′UTR F	CTCGAGCTTCTCGGCTTGGATCCTG
SRC/psi-2 3′UTR R	GTTTAAACATGTCGTGGCCAGAGTTGAC
SRC/psi-2 3′UTRM1 F	CCCGTACTTTGTCCCGTGGAAGGGAAATTCCTGGCCC
SRC/psi-2 3′UTRM1 R	CCACGGGACAAAGTACGGGGAAGGAGGCCAG
SRC/psi-2 3′UTRM2 F	GTCCCCCAAACATGTTGTACATTTCACCATGGCCCCC
SRC/psi-2 3′UTRM2 R	AATGTACAACATGTTTGGGGGACGGAGCGGG
SNAI2/psi-2 3′UTR F	CTCGAGGTGACGCATCAATGTTTACTCG
SNAI2/psi-2 3′UTR R	GTTTAAACTTTTCTTGTTAACAAAGAATTC
KIF2A/psi-2 3′UTR F	CTCGAGACCGGCATTTGCTGCTAAAG
KIF2A/psi-2 3′UTR R	GTTTAAACCAGAATTTCATTCGTTTTTATTAT

Supplementary Table S2: Antibody information and primer sequences for the ChIP assay

ChIP antibodies

Primary antibody	Species	Clonality	Source	Dilution
AR	Rabbit	Monoclonal	Epitomics (#3184-1)	1/50
FOXA1	Rabbit	Polyclonal	Abcam (ab23738)	1/50
OCT1	Rabbit	Polyclonal	Novus (NB100-91899)	1/50
GAPDH	Mouse	Monoclonal	Novus (NB300-221)	1/50
IgG	Rabbit		Santa Cruz (sc-2027)	1/50
IgG	Mouse		Santa Cruz (sc-2343)	1/50

ChIP primers

Site	5′–3′
203ARE1 F	GAAGAGAGGTGGGTTCTTCTTG
203ARE1 R	GGGATGGAAGTGGGCATAG
203ARE2 F	CCTTTCTGATTCCCTTTCCA
203ARE2 R	AGTGCTCAGGAGCCAAGGT
203ARE3 F	CACTAATGGCTCCAGACTTGG
203ARE3 R	GCCGGTCCTACCCACTTAG

Supplementary Table S3: Primer sequences for the qRT-PCR

Gene	5′–3′
SRC F	CCAGGCTGAGGAGTGGTATT
SRC R	TTCGTGGTCTCAGTTTCTCG
SNAI2 F	TGGTTGCTTCAAGGACACAT
SNAI2 R	GCAGATGAGCCCTCAGATTT
KIF2A F	CATTGATTTGGCTGGAAATG
KIF2A R	AAGGCTCTGATGCACTCCTT
GAPDH F	CCAGTAGAGGCAGGGATGAT
GAPDH R	CTTTCATTGTCTTTTCCGCC

Supplementary Table S4: Antibody information for Western blotting

Primary antibody	Clonality	Source	Dilution	Secondary antibody	Source	Dilution
SRC	Polyclonal	Cell Signaling (#2109)	1/2000	anti-rabbit IgG	Jackson Labs	1/5000
SNAI2	Polyclonal	Cell Signaling (#9585)	1/500	anti-rabbit IgG	Jackson Labs	1/5000
KIF2A	Polyclonal	Abcam (ab37005)	1/1000	anti-rabbit IgG	Jackson Labs	1/5000
GAPDH	Monoclonal	Novus (NB300-221)	1/1000	anti-mouse IgG	Jackson Labs	1/5000
β-actin	Polyclonal	GeneTex (GTX109639)	1/1000	anti-rabbit IgG	Jackson Labs	1/20,000
